# 12 Tips for Engaging Medical Students in Health Communications

**DOI:** 10.15694/mep.2021.000048.1

**Published:** 2021-02-16

**Authors:** Nasreen S Quadri, Beth K Thielen, Renee Crichlow, Michelle Rheault, Emily K Vraga, Elisia L Cohen, Serin Edwin Erayil, Elizabeth A Gulleen, Jonathan P Braman, Kristina Krohn

**Affiliations:** 1 University of Minnesota Medical School; 2 Hubbard School of Journalism and Mass Communication; 3 Fred Hutchinson Cancer Research Center

**Keywords:** Medical Communication, Social Media, COVID-19, Medical Education, Communication skills, Health Promotion, Twitter

## Abstract

This article was migrated. The article was marked as recommended.

The proliferation of misinformation during the COVID-19 pandemic provides a clear example of the harms that can occur when medical professionals do not engage with the public regarding health topics. To address this need for accessible, accurate medical information, we taught medical students a COVID-19-specific curriculum tailored to sharing this information with the lay public via social media. Through active learning, students developed their understanding of disease-specific pathophysiology, prevention techniques, treatments, and public health interventions while practicing new skills in public communication as health professionals. After two cohorts completed the course, students’ high-quality medical information about COVID-19 reached >100,000 viewers. To further broaden the impact, we shared the course curriculum through the Association of American Medical College (AAMC) iCollaborative. This curriculum provides a model for future engagement of medical students in health communication with lay audiences.

## Introduction

To meet the public’s need for truthful, understandable health information, health professionals must meet people where they are; currently, the public is highly engaged on social media. The power of social media lies in the formation of relationships and the ability to amplify others’ messages. As a trusted source of information, health care professionals can act as important conduits for sharing and amplifying accurate official public health information ( Hesse *et al.*, 2005; Kwon *et al.*, 2015).

Medical students occupy a unique space in the United States healthcare ecosystem. They have foundational knowledge of diseases and pandemics from the medical school curriculum and yet are not certified for independent practice. Many students have grown up in the digital era, which equips them with skills to lead in the social media landscape. The displacement of medical students from clinical learning experiences due to the COVID-19 pandemic presented a unique opportunity to use the virtual learning environment to equip students with new skills to provide high-quality medical information to the lay public via social media. Students’ medical communications broadly helped the medical profession better serve our patients both inside and outside of our exam rooms.

In this setting, a group of physician medical educators from the University of Minnesota Medical School created an online course called “COVID-19: Outbreaks and the Media” as we briefly described in Quadri *et al.* (2020a). The aim of the course was to teach students about COVID-19 “in a manner that facilitates rigorous evaluation of the evolving sources of information about the disease and to engage the students in public service to the medical profession, and our patients, by amplifying high-quality information about COVID-19 on social media” ( Quadri *et al.*, 2020a).

The lessons of designing health messages for the general public are applicable to almost any health condition. By addressing the following themes through these 12 tips, we offer a model for active learning that can facilitate a deeper knowledge of the chosen medical topic and teach students how to engage with a lay audience to better inform the public about any area of health. In our experience, students are unable to fully participate in the communication exercises without combining these skills with mastery of the chosen medical topic. While class discussions and lectures were essential, the primary focus of the course was for students to study and create their health communication messages independently, returning to a group setting for instruction and feedback. Based on this experience, we would like to share with you 12 tips for engaging medical students as important medical communicators through social media during the time of COVID-19 and promoting health in all aspects through social media well into the future.

## 12 Tips for Engaging Medical Students in Health Communications through 4 Themes

### Theme 1: Gain Comfort with Social Media

#### 1. Teach Important Digital Communication Skills through Simple Introductions

The very first assignment for students in our course was to create a video introduction on Flipgrid, a platform for video and voice recording freely available to educators. We chose Flipgrid to teach basic videography and familiarize students with the platform as we planned to use it again in more complicated assignments in the course. In creating their video introductions followed by in class feedback, students learned how to create high-quality, professional-appearing videos from remote settings without needing expensive equipment by considering lighting, framing, background, and audio optimization.

#### 2. Make it Social (and Positive) from the Beginning

We chose Flipgrid as it is easy to use and encourages students to provide each other with interactive feedback. During the first class, students shared how they created their introduction videos and how they would make their videos better in the future. We facilitated this discussion by providing examples including sharing “bloopers”, “outtakes” and best practices (of our own and demos found online). Having an initial low stakes conversation allowed the students to form relationships with others in the class to improve feedback, which they gave each other on all assignments during the course.

#### 3. Provide a Safe Space for First Drafts

We encouraged students to present first drafts in closed discussion rooms or through class assignments. Students appreciated being able to create a first draft and get feedback on that draft before posting anything on public social media accounts.

### Theme 2: Best Practices of Teaching Health Communication

#### 4. Identify the Audience

Health professionals need to identify their target audience for social media. For example, students may want to get quality COVID-19 information to people in their hometowns, or to a subpopulation like teenagers, or to a specific social group. Before delving into writing, videotaping, recording audio, or crafting an infographic, students must determine who they want to reach with their message.

#### 5. Identify the Area of Interest

Encourage students to take the pulse of their professional identity and passions.

• What do they actually want to do as a professional in general?
• What do they want to share now specifically?
• Who do they want to be perceived as? Now and in the future.
• What is their unique contribution?

Students may want to focus on a specific medical topic or patient demographic. In our course on COVID-19, students chose to focus on very different areas related to the disease, such as amplification of racism during the pandemic, the effects of stay at home orders on LGBTQ youth, and addressing the concerns of pregnant women in the time of COVID.

#### 6. Stop Burying the Lede

Medical students and health care providers are often taught to communicate all of the supportive details before getting to the main point. However, that is not how most people hear or read online, especially during the pandemic when there is an unprecedented volume of new information. They look for the title or the topic; the “lede” in journalistic parlance. The “lede” is the hook at the very beginning of a newspaper article or a nonfiction book where the author tells the reader what they are going to share in the rest of the publication. On social media, the “lede” might be the entire Tweet.

We used the World Health Organization’s communication training slides and the Centers for Disease Control and Prevention’s Crisis, Emergency, and Risk Communication Manual as resources for identifying important techniques for teaching health professionals the best practices in communication with the lay audience (World Health Organization, no date; Centers for Disease Control and Prevention, 2014). We taught students to identify a communication objective to focus on and then to stick to three or fewer talking points. Depending on the medium, the main point or one main talking point may be sufficient.

#### 7. Create a Social Media Account

Once students identify their target audience and their area of interest, then you can either assign a specific social media platform or have students choose the social media channel that is the most appropriate for their audience and aligns with their areas of interest. As students create an account they should think about the following questions:

• What should a professional page look like?
• What name to choose?
• What is your target subject/audience? How is it best to reach them?
• What medium is appropriate for their goals?

In this course, Twitter was chosen as the social media platform of choice because it has gained traction as a tool for medical education in recent years and because of the low threshold to join conversations with established physicians, thought leaders, and members of the public ( Forgie, Duff and Ross, 2012; Colbert et al., 2018; Jaffe et al., 2020). Analysis of Twitter during the H1N1 outbreak established that tweets can be used for knowledge translation work, allowing medical and public health authorities to respond to real-time concerns ( Chew and Eysenbach, 2010). We specifically created assignments to advance students through “Modified Bloom’s Taxonomy of Social Media” as shared by Jaffe et al.(2020) in Academic Medicine, starting with consuming social media and evaluating it, then promoting quality information before engaging in discussions on social media with personal content creation.

### Theme 3: Assess the Literature Landscape

#### 8. Narrow the Field to Focus to One Topic

Medical students and physicians, in our experience, express discomfort in sharing publicly about a medical topic in the absence of mastering it. As they studied this one topic in depth, they were better able to identify high-quality methods for sharing the information that they learned. Creating blogs, tweets, and infographics deepened their comfort and knowledge on the topic. To help students pick an appropriate topic in the early part of the course, we actively discussed and shared COVID-19-related news from health media, social media, and the scientific literature. We started by providing broad overviews and introductions to different topics and then encouraged students to search the news, social media, and scientific literature to find a specific topic within the broader domains of COVID-19 (for example, testing; telemedicine; convalescent plasma therapy; impact on minority groups; vaccination, etc.) to study with the intent of creating content about this topic to share with a lay audience. This strategy could be easily adapted to different medical topics.

#### 9. Optimize Students’ Ability to Find Literature of All Types

In order to write, the students needed to find quality information. This course, and the online nature of it, provided the opportunity for students to improve their ability to quickly search PubMed and Google. When students had difficulty finding materials, it was easy to ask students to screen share with a teacher or librarian as the student searched the web or PubMed. Students were then better able to search on their own than if we had only demonstrated how to search. To ensure that students were able to find the best scientific articles for their topics, students captured videos of themselves actively demonstrating their use of advanced search options in PubMed. Students then shared these videos on Flipgrid, where classmates provided feedback. They learned how to problem solve with their peers when they had challenges.

#### 10. Enhance Critical Evaluation of Literature

Students need to optimize their ability to determine if a given website, tweet, or scientific article is credible and worth sharing. We discussed bias and traditional means of evaluating an article’s credibility, including understanding the methods and results that an article uses. In particular, the difference between relative and absolute risk is often a tricky concept that authors may manipulate to support different conclusions.

We also introduced students to skills utilized by professional fact-checkers, people who make their living trying to tell the truth from fiction about a topic they may know very little about. They read the study by Wineburg and McGrew that described how fact-checkers assess the quality of information provided online, identifying several specific techniques that students can quickly employ ( Wineburg and McGrew, 2017). The most important technique introduced was “lateral reading”. In contrast to fact-checkers, most individuals read a text online from the beginning to the end, from the top to the bottom, i.e. vertically. Professional fact-checkers look at an article and immediately start opening new tabs in the web browser (moving laterally on the screen rather than down the page) to investigate the author’s background, organization’s histories, and following funding sources before they returned to the original article and then read the article’s contents. They use the power of the internet to obtain external information first to determine whether the article is worth reading further before implementing traditional methods for verifying the validity of the article.

We encouraged students to combine these “fact-checker techniques” with traditional methods for ensuring the quality of an article or website. Once again, the virtual platform allowed for screen sharing to enable demonstration of how this technique can be used. Ultimately, students demonstrated their “lateral reading” skills through screen captured video submission on Flipgrid while learning about their medical topic. Before encouraging students to share content publicly, we wanted to ensure they could be critical of the quality and the bias of published information.

### Theme 4: Bring it all together

#### 11. Amplify One Topic in Several Communication Formats

After students demonstrated proficiency in consuming and evaluating media about their medical topic and created a communication plan, they created a variety of social media communications. Students used their communication objective and talking points as the foundation for writing a blog post. Students’ blog posts were posted on A Blog of Medical Discourse ( Quadri et al., 2020b). The student’s blog content was then adapted to new communication modalities such as infographics, video “sound bites” and their final product of a Tweetorial, a series of Tweets linked together to make a tutorial on Twitter. We discussed which aspects made a given Tweet worth repeating from the health communication literature, including visual elements such as infographics or videos; engaging wording, direct messaging, Tweets that felt personally relevant, or Tweets that engaged people in conversations ( Vos and Buckner, 2015; Vos et al., 2018).

#### 12. Evaluating Performance Through Metrics

We taught students how to evaluate their impact. The final assignment in our class was to have students share their Twitter Analytics page as a means of reflecting on the reach of their social media posts. Students then discussed techniques they could have employed to expand that reach to a broader audience based on their experiences throughout the course.

## Conclusions

We implemented this course with two groups of students and received feedback from 14 of the total of 20 students who took the course. The majority of these students (79%) endorsed the course helping further their knowledge of COVID-19 while learning skills to identify high-quality sources of information. Most students (79%) also reported increased comfort engaging with the general public as a medical provider. The cumulative Twitter analytics from two sessions of the class revealed > 100,000 Twitter impressions, relaying engagement with a significant number of Twitter users. A Twitter impression reflects the number of times a Tweet goes across a user’s screen and is theoretically read by that person. Half of the students in each class have continued to be actively posting on their professional Twitter accounts since completing the course.

Our evaluation demonstrates that teaching students how to share health information publicly through social media can help students gain mastery over a new medical topic, while also performing a public service to our profession and our patients. This active learning format can be adapted for other areas of medical focus to help improve the health of the population where our students live and study. Our curriculum can be openly accessed on our website ( https://z.umn.edu/COVIDMedia) and the iCollaborative website of AAMC to be further adapted for other institutions ( Krohn *et al.*, 2020).

Our experience teaching health communication during the COVID-19 pandemic exemplifies how medical students can learn these skills in a way that is effective for reaching the public. We believe that integrating health communication training into the formal medical education curriculum will better meet the evolving needs of our patients and our students.

## Take Home Messages

• Medical students can, and should, be taught how to effectively share health information with lay audiences through social media
• Through these 12 Tips through 4 Themes, educators can learn how to design their own courses for teaching medical students important communication skills
• We must help students:
  • gain comfort using social media as a professional
  • learn the best practices of health communication
  • optimize their ability to assess the quality of the information found from a variety of sources
  • practice sharing medical information through a variety of media formats

## Notes On Contributors

**Nasreen S Quadri**, MD completed her year as Global Medicine Chief Resident at the University of Minnesota and now is a Med/Peds hospitalist at Abbott Northwestern Hospital, part of Allina Health in Minneapolis, Minnesota. ORCID ID: https://orcid.org/0000-0002-3236-0479

**Beth K Thielen**, MD, PhD completed her fellowship in adult and pediatric infectious diseases and is an Assistant Professor of Pediatrics Infectious Diseases and Immunology at the University of Minnesota. Her laboratory studies the pathogenesis of respiratory viruses including influenza and respiratory syncytial virus. ORCID ID: https://orcid.org/0000-0001-5922-0241

**Renee Crichlow**, MD is the holder of the Mac Baird Endowed Chair for Family Medicine Advocacy and Policy at the University of Minnesota Family Medicine and Community Health and teaches and practices full-spectrum family medicine. ORCID ID: https://orcid.org/0000-0002-6284-1679

**Michelle Rheault**, MD a pediatric nephrologist, has taught many medical students and residents how to use social media to improve the health of kidneys around the world. ORCID ID: https://orcid.org/0000-0003-2494-3970

**Emily Vraga**, PhD is an associate professor in the Hubbard School of Journalism and Mass Communication at the University of Minnesota. Her research tests methods to identify and correct health misinformation on social media, to limit biased processing of news messages, and to encourage attention to more diverse content online. ORCID ID: https://orcid.org/0000-0002-3016-3869

**Elisia L Cohen**, PhD the Director of the Hubbard School of Journalism and Mass Communication at the University of Minnesota is an expert in health communication education and media intervention design and dissemination. ORCID ID: https://orcid.org/0000-0003-2039-8936

**Serin Edwin Erayil**, MD is the Chief Infectious Diseases Fellow at the University of Minnesota and studies the epidemiology of Candidemia as her primary area of interest. ORCID ID: https://orcid.org/0000-0002-4912-4923

**Elizabeth A Gulleen**, MD is an infectious disease physician and Research Associate at the Fred Hutchinson Cancer Research Center where she studies the treatment of cancer-related infections in low-and-middle-income countries. ORCID ID: https://orcid.org/0000-0003-0035-5015

**Jonathan P Braman**, MD, the former Orthopedic Residency Program Director, teaches medical students and the general public about shoulders through Twitter. ORCID ID: https://orcid.org/0000-0002-0319-1311

**Kristina Krohn**, MD is a Med/Peds Hospitalist at the University of Minnesota. Since completing the Stanford University Global Health and Media Fellowship in 2013 she has been a staunch advocate of empowering physicians to share more about medicine and health with the general public. ORCID ID: https://orcid.org/0000-0001-6116-7128
