# Supplementary material for: 12 Tips for Engaging Medical Students in Health Communications
Source: MedEdPublish (2016). 2021 Feb 16;10:48. [Version 1] doi: 10.15694/mep.2021.000048.1 (PMC10939613; doi:10.15694/mep.2021.000048.1)
Supplement: Peer review history

# Reviewer report: Reviewer response for version 1

**DOI:** 10.21956/mep.18885.r26959

**Published:** 2021-06-03

**Authors:** Ken Masters

**Affiliations:** 1 Sultan Qaboos University

This review has been migrated. The reviewer awarded 3 stars out of 5 An interesting 12-tips paper on engaging medical students in health communications. I am particularly pleased that this course deals with teaching medical students the use of social media in communication skills. (It’s a personal bugbear of mine that almost all courses on medical communication deal with face-to-face, one-to-one communication, as if we still live in the 19th century, and electronic communication with patients and the public, in general, does not exist).Two issues that I would like to see addressed:• As this course was put together to meet a particular need, it is understandable that the paper focuses on the practicalities of the particular situation. As a result, there is some theoretical background missing, and I would like to have seen some reference to patient engagement with electronic communication and, in particular, with social media (with reference to the concept of the e-patient). This would enhance the paper by demonstrating that the goal is _engagement_, rather than _information broadcast_.• In addition, I would, like to see a little more detail on some of the tips, perhaps with screenshots/links. This would add value to the paper for novices (the prime target for a 12-tips paper) to get them started on their own projects.So, a useful and encouraging read. I look forward to Version 2 in which these issues are addressed.

Reviewer Expertise:

NA

No decision status is available

# Reviewer report: Reviewer response for version 1

**DOI:** 10.21956/mep.18885.r26960

**Published:** 2021-02-18

**Authors:** BALAJI ARUMUGAM

**Affiliations:** 1 TAGORE MEDICAL COLLEGE AND HOSPITAL

This review has been migrated. The reviewer awarded 4 stars out of 5 Kudos to the authors for publishing such a wonderful practical tips on health communication curriculum for the medical students. The 4-week curriculum course designed to engage medical students in understanding the COVID-19 epidemic, track its progression using reliable sources and empower them to engage with the community via social media. Congratulations to the course coordinator - Kristina Krohn and the team. This article demonstrates that the implementation of how to share health information publicly through social media which helped students to gain mastery over a new medical topic, while also performing a public service to our profession and our patients. Its novel and very important need of the hour for all the medical professionals as everything is getting digitalized in covid situation. Once again thanks and congrats to the authors.

Reviewer Expertise:

NA

No decision status is available

# Reviewer report: Reviewer response for version 1

**DOI:** 10.21956/mep.18885.r26961

**Published:** 2021-02-17

**Authors:** Dawn Dillman

This review has been migrated. The reviewer awarded 4 stars out of 5 This is a description of a novel curriculum to teach medical students how to effectively use social media use to communicate about health issues to the general public. It is a well thought out curriculum using multiple modalities and both expert and peer-to-peer teaching. Twitter views for the final outcome measure is a clever choice as an objective measure of success of the curriculum. I wonder if the article would engender more interest with either social media or Twitter in the title as “Health Communication” is a very broad statement that somewhat buried the lede. Thank you for sharing this interesting work!

Reviewer Expertise:

NA

No decision status is available
